# Preoperative measurement of cutaneous melanoma and nevi thickness with photoacoustic imaging

**DOI:** 10.1117/1.JMI.5.1.015004

**Published:** 2018-02-13

**Authors:** Aedán Breathnach, Elizabeth Concannon, Jemima J. Dorairaj, Shazrinizam Shaharan, James McGrath, Jithin Jose, Jack L. Kelly, Martin J. Leahy

**Affiliations:** a National University of Ireland (NUI), Tissue Optics and Microcirculation Imaging Facility, National Biophotonics and Imaging Platform, Galway, Ireland; b University Hospital Galway, University College Hospital Galway, Department of Plastic and Reconstructive Surgery, Ireland; c FUJIFILM Visualsonics Inc., Amsterdam, The Netherlands; d Royal College of Surgeons (RCSI), National Biophotonics and Imaging Platform, Dublin, Ireland

**Keywords:** biopsy, Breslow depth, spectral unmixing, histology, staging

## Abstract

Photoacoustic imaging (PAI) is an emerging biomedical imaging technology, which can potentially be used in the clinic to preoperatively measure melanoma thickness and guide biopsy depth and sample location. We recruited 27 patients with pigmented cutaneous lesions suspicious for melanoma to test the feasibility of a handheld linear-array photoacoustic probe in imaging lesion architecture and measuring tumor depth. The probe was assessed in terms of measurement accuracy, image quality, and ease of application. Photoacoustic scans included single wavelength, spectral unmixing, and three-dimensional (3-D) scans. The photoacoustically measured lesion thickness gave a high correlation with the histological thickness measured from resected surgical samples (r=0.99, P<0.001 for melanomas, r=0.98, P<0.001 for nevi). Thickness measurements were possible for 23 of 26 cases for nevi and all (6) cases for melanoma. Our results show that handheld, linear-array PAI is highly reliable in measuring cutaneous lesion thickness *in vivo*, and can potentially be used to inform biopsy procedure and improve patient management.

## 1 Introduction

Melanoma is a tumor that results from the malignant transformation of melanocytes and is the most lethal form of skin cancer. Despite accounting for <5% of all skin cancers, it is responsible for 75% of skin-cancer-related deaths.^1^^,^^2^ Early diagnosis and treatment is essential, with a 98% survival rate for cases discovered before the tumor metastasizes to the sentinel lymph nodes and organs. The histopathologically measured melanoma thickness, known as the Breslow thickness, is the most important clinical indicator for melanoma staging, guiding treatment, and determining prognosis.

Although the recommended technique for diagnosing cutaneous melanoma is excisional biopsy with narrow margins, partial biopsy techniques, such as punch and tangential biopsies, are routinely used to diagnose suspect lesions, particularly in cosmetically sensitive areas, with up to 27% of melanomas being diagnosed with partial biopsy.^3^^,^^4^ Partial biopsies are associated with an increased risk of inaccurate histopathologic measurement of tumor thickness and misdiagnosis, due to undersampling of the primary lesion.^5^ Microstaging inaccuracies for melanoma have been reported in 16% to 43% of nonexcisional biopsy techniques.^5^^,^^6^ Current guidelines suggest that the most irregular and pigmented part of a suspect lesion is the most favorable for biopsy; however, this does not always correspond to the most histologically advanced area of the lesion,^5^^,^^7^^,^^8^ and thus the full lesion thickness may be unavailable to the pathologist. In cases where undersampling occurs, surgeons can be faced with a choice of planning a full surgical excision based on a provisional Breslow thickness, in the hope that it represents the thickest portion of the lesion, or to perform a repeat biopsy to obtain the full lesion thickness prior to definitive surgery.^9^ Thus, a preoperative, noninvasive measurement of tumor thickness could guide the surgeon in determining biopsy depth and sample location and could improve patient management and eliminate the need for additional biopsies.

Many noninvasive imaging modalities have been used for the *in vivo* assessment of melanoma and benign skin lesions, but many have significant limitations. Dermoscopy^10^ is routinely used to examine subsurface tumor characteristics and pigmentation, and has aided the diagnosis of melanoma. However, its penetration depth is limited by optical diffusion, and it cannot image beyond the papillary dermis. Other optical imaging methods, such as optical coherence tomography,^11^ confocal microscopy,^12^^,^^13^ and two-photon microscopy,^14^ while providing good contrast and resolution, are likewise depth limited and do not have sufficient penetration to determine melanoma depth. High-frequency ultrasound (US) can maintain good resolution at penetration depths greater than optical imaging but it has poor contrast as the difference in acoustic impedance between melanoma and the surrounding tissue is low.^15^ Magnetic resonance imaging and positron emission tomography^16^^,^^17^ have been used in the assessment of melanoma, however, they have poor resolution in the skin, are expensive, and are not routinely available for clinical dermatological imaging.

Photoacoustic imaging (PAI) is a noninvasive biomedical imaging modality which combines the advantages of optical and US imaging, namely high optical absorption contrast with high US resolution, for deep tissue imaging in the diffusive regime, while minimizing their disadvantages.^18^ In PAI, a pulsed source of electromagnetic energy, typically a short-pulsed laser, is used to heat biological tissue through optical absorption. The resultant thermoelastic expansion generates an acoustic wave that can be detected at the tissue surface in the same way as conventional US imaging. PAI derives its means of contrast from optical absorption and is thus sensitive to endogenous biological absorbers, such as melanin and hemoglobin, allowing it to image melanin distribution within the skin and pigmented skin lesions, as well as their associated microvasculature networks.^19^^,^^20^ By varying the laser wavelength, PAI can acquire functional information such as blood oxygen saturation and hemoglobin concentration measuring angiogenesis—a hallmark of cancer. Moreover, since US scattering is 2 to 3 orders of magnitude weaker in biological tissue than optical scattering, PAI can image beyond the optical ballistic regime (∼1  mm in soft tissue) while maintaining high spatial resolution.

Several preclinical studies, employing PAI systems with various scanning configurations, have demonstrated its effectiveness in assessing the axial and lateral extent of cutaneous skin lesions, including melanoma, and their associated microvasculature.^21^^–^^23^ PAI systems with handheld, linear-array probes are most likely to succeed in clinical applications due to their high frame rate, field of view, and ease of application.^24^^,^^25^ Furthermore, linear-array systems allow for the simultaneous coregistration of PA and B-mode US images, which combine the optical absorption contrast and functional information of PAI with the structural imaging capabilities of US.^26^

In this study, we introduce a high-frequency, handheld linear-array PAI system to the clinic to test its feasibility, in terms of ease of application, image quality, scanning time, and accuracy in measuring pigmented skin lesion thickness *in vivo*. To this end, patients with cutaneous lesions suspicious for melanoma were recruited to undergo a preoperative PA scan. PA lesion thickness measurements were compared with the histologically determined thickness measured from resected surgical samples.

## 2 Materials and Methods

After ethical approval from our institutional review board, 27 patients with pigmented cutaneous lesions that aroused a clinical suspicion of melanoma were recruited to undergo PAI. All patients were informed and gave consent before participation. During scanning, patients were required to wear laser safety goggles for protection from laser radiation.

**Fig. 1:**
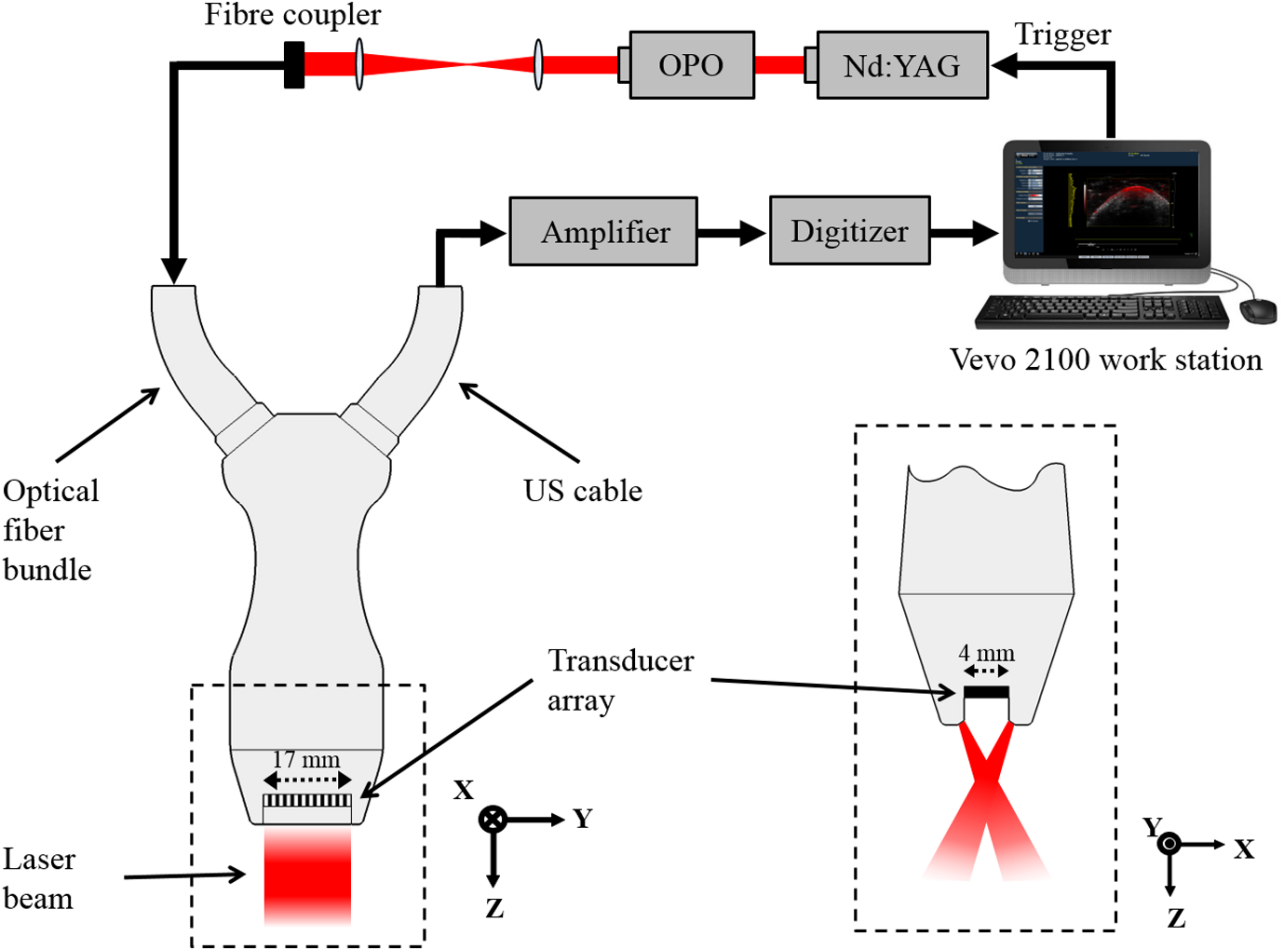
PAI system used to scan pigmented skin lesions. (Left) Schematic of PAI system showing laser source, signal processing procedure, and linear-array PA probe (viewed from elevational direction). (Right) View of the PA probe head (enclosed by dashed box on left) showing crossed laser beam geometry (lateral direction).

The PA linear-array probe (LZ 550, VisualSonics Inc.) was composed of 256 acoustic transducers, with a central detection frequency of 40 MHz (bandwidth 55%), and surface area of 3  mm×14  mm, with a large acceptance angle allowing the entire section of most lesions to be assessed in the imaging plane. The use of a linear array generates inherently coregistered PA and US images in real time, which provide a complementary fusion of US structure with the functional and molecular information of PA images.^27^ Pulsed laser light was delivered to the tissue through two planar light bars (Fig. 1), situated on either side of the transducer array, and focused at a distance of 7 mm from the transducer surface. The beam angle was 30 deg with respect to the imaging plane. A tunable optical parametric oscillator (OPO, 680 to 970 nm, Opotek Inc.) driven by a frequency-doubled Nd:YAG laser (20 Hz repetition rate, 5 frames per second) provided the laser light source and was coupled to an optical fiber bundle and incorporated to the PA probe. The detected PA and US signals were sent through a cable to an imaging station (Vevo LAZR, VisualSonics Inc.), where a delay-and-sum beam forming algorithm was used to reconstruct the coregistered PA and US images and display them in real time. Furthermore, by scanning the linear-array probe along a one-dimensional stepper motor, three-dimensional (3-D) images of skin lesion morphology could be formed by taking a two-dimensional (2-D) scan at regular intervals.^28^ The frame density of 3-D scans was set to 290 frames per scan length, which was varied depending on the lesion diameter. A single 3-D scan took ∼3  min to acquire, and during scanning the patient was instructed to keep still to prevent movement artifacts.

The PA probe was coupled to the skin with US gel and scanned laterally across the lesion in several scanning directions to assess its full volume. Single-wavelength PA scans were taken at 680 nm. Care was taken to ensure that the PA probe was placed perpendicular to the skin surface to maximize the amplitude of the detected PA signal. The frame with the deepest portion of the lesion was selected, and the maximum lesion thickness was measured from the top of the epidermis to the deepest discernible lower boundary, as distinguished by high optical absorption.

In addition to single-wavelength PA scans, an imaging technique known as spectral unmixing (SU)^29^ was employed. SU uses multiwavelength PA scans to produce a PA image that maps the spatial distribution of a selected biological absorber, in this case melanin and melanin-containing cells (e.g., melanocytes and basal keratinocytes), by means of their distinct optical absorption spectrum within the near-infrared wavelength region. The SU scanning procedure consisted of taking a spectroscopic scan of the most pigmented part of the lesion over the wavelength range of 680 to 970 nm in steps of 10 nm, and a subsequent multiwavelength scan where the laser sequentially switched between 5 wavelengths (680, 700, 750, 850, and 900 nm). The multiwavelength scan was used to produce a PA image that exclusively plots regions of absorption, which matched the absorption spectrum of the lesion. Since SU separates the absorption signature of melanin-containing cells from other endogenous chromophores in the tissue, it was hypothesized that it may provide a more accurate measure of lesion thickness. To test this, SU thickness measurements were compared with thickness measurements from single-wavelength PA scans. Furthermore, SU scans were used to track the extent of lesion growth along the epithelium in skin adnexa, such as hair follicles and sweat glands, in cases where said measurements were reported by histology. Adnexal depth was measured vertically from the lesion surface to the deepest adnexal structure connected to the primary lesion. On average, the full SU scanning procedure took ∼7  min.

The maximum PA lesion thickness, and the depth of lesion extension in the skin adnexa measured with SU, was compared with the histological measurements from resected surgical specimens. PA thickness measurements were made blinded to results of the histological thickness. Correlation between PA measurements and histological measurements was determined by means of the correlation coefficient (CC) obtained from a linear regressive fit. The percentage error between the PA measurements and the histological measurements was equal to the difference between the PA and histology thickness divided by the histological thickness (100×). Two-tailed t-tests were employed to test the statistical significance between results.

## 3 Results

A total of 32 pigmented cutaneous lesions were scanned on 27 patients, with histology confirming a diagnosis of melanoma in six cases. All patients displayed primary tumors, located on the trunk, extremity, head, or neck (lesions immediately adjacent to the eyes were excluded due to laser safety risk), without visible ulceration, which allowed application of the probe. Benign lesions included compound and intradermal melanocytic nevi (8 and 5, respectively), seborrheic keratosis (3), and dysplastic nevi (4), whereas malignant tumors included *in situ* (3) and invasive melanomas (3). Benign lesions were very ill-defined in some cases, with lower structural boundaries being invisible in three cases. Melanoma lower boundaries were determined in all cases.

**Fig. 2:**
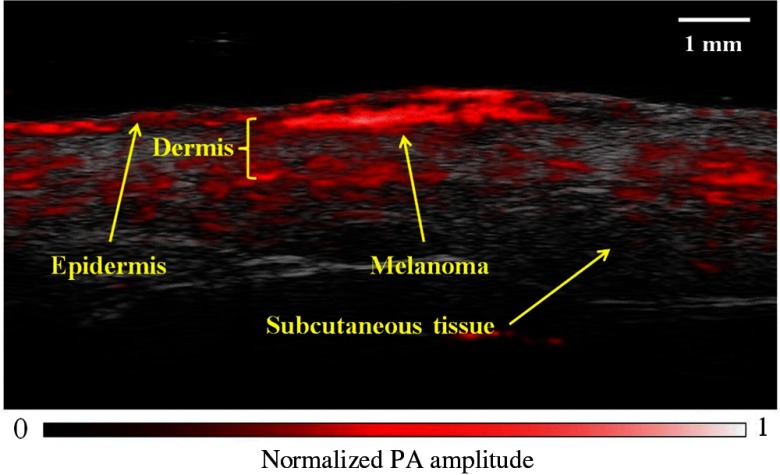
Coregistered PAI image of *in situ* melanoma on upper left extremity. Handheld linear-array based PAI was able to image pigmented lesion and skin architecture with high contrast, speed, and resolution.

**Fig. 3:**
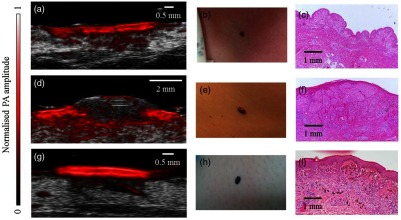
Coregistered linear-array based PAI of melanoma and benign skin lesions. (a) Coregistered PAI image of *in situ* melanoma with a PA depth of 0.3 mm. (b) *In situ* melanoma on chest. (c) Histology of excised melanoma, with Breslow depth of 0.15 mm. Original magnification ×4. (d) Coregistered PAI image of invasive melanoma on back, showing signs of dermal invasion, with a PA depth of 1.85 mm. (e) Invasive melanoma on back. (f) Histology after full excision, with a Breslow depth of 1.6 mm and Clarke level IV. Original magnification ×4. (g) SU image of dysplastic nevus showing adnexal extension, with a PA depth of 0.36 mm and adnexal depth of 1.6 mm. (h) Dysplastic nevus on lower left extremity. (i) Histology of excised nevus, with a histological depth of 0.39 mm and adnexal depth of 2.08 mm. Original magnification ×4.

On the PA images, lesions boundaries could be clearly distinguished from the surrounding soft tissue, with well-demarcated lower boundaries distinguished by high optical absorption. Skin layers, such as the epidermis, dermis, and subcutaneous tissue could be identified (Fig. 2). Superficial lesions, such as compound nevi and thin seborrheic keratoses, appeared as symmetrical masses with well-defined regular borders with no visible presence in the dermis. *In situ* melanomas had more uneven boundaries but were not seen to invade past the basal layer of the epidermis, as confirmed by histopathology [Fig. 3(a)]. Invasive lesions, such as intradermal nevi and invasive melanoma, generally had irregular borders, due to uneven proliferations of melanoma cells and melanocytes, and could be distinguished from superficial lesions through growth of the primary lesion through the dermal–epidermal junction [Fig. 3(d)]. SU scans showed the presence of melanin-containing cells extending through skin appendages connected to the primary lesion [Fig. 3(g)].

**Fig. 4:**
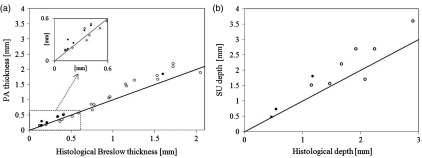
Correlation between *in vivo* measurements of melanomas (black circles) and benign lesions (clear circles) and histological measurements from biopsied samples (black line shows 1:1 agreement for PAI measurements and histological measurements). (a) Plot of primary lesion thickness measured with PAI against histological thickness. (b) Plot of adnexal tumor extension measured with SU against histological depths.

Statistical analysis and plots of *in vivo* thickness measurements against histological thickness are shown in Table 1 and Fig. 4. In Fig. 4, the slope of the linear fit indicates the sensitivity of the imaging system in estimating lesion depth. For PA and SU, the slope was found to be >1.1, indicating a tendency to over-estimate lesion thickness.

**Table 1:** CC, median percentage errors, and statistical significance of PAI and SU measurements of lesion thickness and adnexal depths, for melanomas and benign lesions.

Imaging modality	N	CC	Mean % error	t-test P value
PA benign	23	0.98	13.4	0.60
		P<0.001		
SU benign	23	0.98	13.1	0.67
		P<0.001		
PE melanoma	6	0.99	22.3	0.76
		P<0.001		
SU melanoma	6	0.99	22.1	0.80
		P<0.001		
SU adnexae	10	0.93	37.8	0.44
		P<0.001		

For benign lesions, PAI and SU gave a high correlation with the histological thickness, with a CC of 0.98 (P<0.001) for both. For cases where an *in vivo* measurement was possible, the mean PA thickness was 0.96 mm (range, 0.14 to 2.2 mm), giving a 13.4% error with the histological thickness of 0.84 mm (0.12 to 2.05 mm), whereas the mean SU thickness was 0.86 mm (0.13 to 2.0 mm), with a percentage error of 13.1%. Benign lesions thickness was overestimated in 69% of cases. For melanomas, a CC of 0.99 (P<0.001) was obtained for both PAI and SU. The mean percentage error for PA was 22.3%, for a mean PA thickness of 0.58 mm (0.17 to 1.85 mm) and mean Breslow thickness of 0.47 mm (0.15 to 1.6 mm). For SU, the mean percentage error was 22.1%, with a mean thickness of 0.56 mm (0.17 to 1.74 mm). Both PA and SU overestimated melanoma thickness in all cases. Adnexal measurements were reported in histology for 12 lesions, with SU depth measurement being possible in 10 cases. The CC between SU adnexal depth and histology was 0.93 (P<0.001). Mean histological depth was 1.56 mm (0.46 to 2.9 mm) compared with a mean SU depth of 2.15 mm (0.47 to 3.6 mm), with a percentage error of 37.8%. Two-tailed t-test analysis yielded no statistically significant differences between results for all measurement groups.

**Fig. 5:**
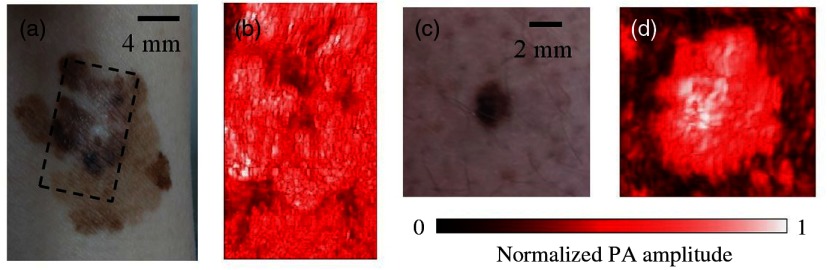
Rendered 3-D PA images of pigmented skin lesions taken using a stepper motor. (a) Large diameter melanoma *in situ* located on ventral aspect of wrist. (b) 3-D PAI scan (area=14  mm×235  mm) of dashed region in (a) showing complex pigmentation pattern. (c) Compound melanocytic nevus located on lower left extremity. (d) 3-D PAI scan (area=4  mm×4  mm) of lesion in (c). 3-D PA scans assessed lesion volume and allowed for the thickest portion to be registered to the surface, which can potentially guide incisional biopsy location and depth.

3-D rendered scans of an *in situ* melanoma and benign lesion are shown in Fig. 5. 3-D PA scans provided a map of the overall lesion architecture, allowing for the thickest point to be identified and correlated to the surface topography, which can be difficult with 2-D images. However, in some cases, 3-D scans had to be discarded due to movement artifacts.

## 4 Discussion

PAI represents a new technique for the *in vivo* evaluation of melanoma and benign skin lesions in dermatology. The main strength of PAI is its ability to image molecular changes at clinically significant depths. In this study, we have demonstrated the feasibility of PAI in measuring the *in vivo* depth of pigmented melanocytic nevi and melanomas on patients in the clinic. The use of a handheld, linear-array PA probe provided an easily applicable means of imaging the entire lesion architecture with high contrast, speed, and resolution.

PAI was found to accurately measure primary lesion thickness, for both melanocytic nevi and melanomas, as evidenced by the high CC obtained. In general, PAI, tended to overestimate lesion thickness compared with the histologically measured thickness. This was expected, as dehydration of the resected samples in the histological sectioning process results in sample shrinkage, which is further compounded by loss of *in vivo* skin tension.^30^ Although care was taken to ensure registration of the PAI measurement location with the histological sections obtained from biopsy, this cannot be guaranteed and could therefore effect measurement correlation. Moreover, unlike histology, handheld B-mode and 3-D scans allow for the entire lesion volume to be assessed, and the deepest portion to be selected for measurement.

Lower lesion boundaries could not be determined for nevi in three cases, possibly due to strong optical attenuation from absorption in highly pigmented lesions or possible suboptimal directional placement of the PA probe.

PAI’s ability to image the skin layers and lesion architecture with high contrast enabled it to distinguish between invasive and superficial lesions by their penetration through the dermal–epidermal boundary. Benign intradermal lesions were identified as such in all cases; however, epidermal lesions were misread as having a dermal presence in two cases due to irregular border profiles. For melanomas, PAI was able to structurally distinguish *in situ* melanomas from invasive melanomas in all cases. In cases where a melanoma diagnosis is made with an undersampled biopsy, preoperative knowledge of lesion penetration through the dermal–epidermal boundary could expedite and inform the treatment process, as this is a critical step in the metastatic development of melanoma.

SU had a slightly lower measurement error for primary lesion thickness; however, since the contrast ratio between the background tissue and pigmented lesions is high in the NIR, its accuracy over single-wavelength PAI at 680 nm was negligible. SU was able to track lesion extension in skin appendages in most cases and can potentially guide the surgical management of adnexal-based malignant tumors.^31^^,^^32^ However, it had a large measurement error due to difficulty ensuring registration between *in vivo* sections and histology, and in the case of hair follicles, melanin presence in hair shaft made it difficult to determine where lesion extension ended.

The high correlation between PAI depth measurements and histopathology allows for the establishment of a linear depth index,^30^ which accounts for measurement errors, such as shrinkage effects. While PAI is more suited to imaging pigmented melanomas (>90% of cases), it can potentially be used to image amelanotic melanomas as they still contain a low concentration of melanin. Our results show that handheld linear-array PAI could help guide biopsy depth and sample location, and thus improve staging and diagnostic accuracy and prevent the need for additional biopsies. Demonstrating the staging potential of PAI in the clinic is important, as it has the potential to diagnose melanoma and other skin cancers with the injection of targeted antibodies coupled with PA sensitive contrast agents.^33^ While PAI shares the same means of detection and associated electronics as US imaging, it can have a considerably higher cost if pulsed nanosecond lasers are employed as the excitation source. However, it is still relatively inexpensive when compared with PET and MRI. PAI systems which employ inexpensive laser diode sources as the excitation means can significantly reduce costs, rendering PAI on the same cost level as US imaging.^34^
